# Uterine-Sparing vNOTES vs. laparoscopic lateral suspension for pelvic organ prolapse repair: a prospective comparative study of feasibility and early outcomes

**DOI:** 10.3389/fsurg.2026.1797662

**Published:** 2026-05-08

**Authors:** Abdurrahman Sengi, Mehmet Nuri Duran, Mayıs Jinda Pekgül, Nurullah Peker

**Affiliations:** 1 Department of Obstetrics and Gynecology, University of Health Sciences Gazi Yaşargil Training and Research Hospital, Diyarbakır, Türkiye; 2 Department of Obstetrics and Gynecology, Ezine State Hospital, Canakkale, Türkiye; 3 Department of Obstetrics and Gynecology, Medical Faculty of Dicle University, Diyarbakır, Türkiye

**Keywords:** laparoscopic lateral suspension, minimally invasive surgery, pelvic organ prolapse, POP-Q, vNOTES

## Abstract

**Background:**

Vaginal natural orifice transluminal endoscopic surgery (vNOTES) represents a minimally invasive uterus-preserving technique for apical prolapse repair. Comparative data with laparoscopic lateral suspension are scarce. This study aimed to evaluate perioperative performance, anatomical correction, and functional outcomes of both approaches.

**Methods:**

In this prospective cohort, 100 women with symptomatic anterior–apical pelvic organ prolapse (POP-Q ≥ II) underwent either vNOTES lateral suspension (*n* = 50) or LLS (*n* = 50). Perioperative metrics, POP-Q parameters, and validated functional questionnaires (POPDI-6, UDI-6, CRADI-8) were recorded preoperatively and at 3 and 6 months. Complications and recurrence were assessed. Radar visualization was used to illustrate POP-Q approximation relative to expected anatomical contours.

**Results:**

Both techniques provided successful anterior–apical correction. Operative time (60.4 ± 11.2 vs. 90.7 ± 8.4 min; *p* < 0.001) and length of hospital stay (1.32 ± 0.59 vs. 1.98 ± 0.65 days; *p* < 0.001) were significantly shorter with vNOTES. POP-Q measurements favored vNOTES at 6 months for Ba and Bp (*p* < 0.001), whereas point C and total vaginal length were comparable. Functional outcomes demonstrated lower POPDI-6 and UDI-6 scores in the vNOTES cohort at both follow-ups (*p* < 0.05). No mesh exposures occurred. Complication rates were low and did not differ significantly. Radar visualization demonstrated more uniform postoperative approximation in the vNOTES group.

**Conclusion:**

Both vNOTES and laparoscopic lateral suspension achieved effective uterus-preserving prolapse repair with favorable short-term anatomical and functional results. vNOTES provided faster perioperative recovery and greater functional symptom improvement without compromising safety. Extended follow-up will clarify durability.

## Introduction

Pelvic organ prolapse (POP) refers to the downward descent of pelvic organs due to dysfunction of the pelvic floor's connective tissue support. POP is a pelvic floor disorder that is frequently observed in older women and can significantly impair quality of life. The prevalence of POP on physical examination has been reported to reach 40%–50%, whereas the symptomatic prevalence is estimated to be approximately 3%–6% (1, 2). While pessaries and pelvic floor muscle training may be appropriate for selected patients, surgery remains the most effective treatment for women with advanced POP or those whose symptoms substantially compromise quality of life (3). Adequate apical support is essential during POP repair, since inadequate apical suspension significantly increases the risk of anterior and posterior compartment recurrence.

Over the past decade, technological advances in minimally invasive pelvic floor surgery have expanded the laparoscopic applicability of apical prolapse repair techniques such as sacrocolpopexy, which has long been considered the gold standard, and have been associated with reduced blood loss, faster recovery, and lower rates of neurovascular complications compared with open surgery. However, laparoscopic sacrocolpopexy requires dissection of the presacral space, which may lead to rare but serious complications such as major vascular injury, hypogastric nerve damage, osteomyelitis, and diskitis, and it also entails a relatively steep learning curve and substantial technical expertise. These considerations have fueled clinical interest in less invasive apical suspension approaches with shorter learning curves, lower vascular risks, and improved cosmetic outcomes. Laparoscopic lateral suspension (LLS) has emerged in this context as an effective minimally invasive alternative particularly for anterior–apical POP repair, allowing uterine preservation, restoring the vaginal axis in a more physiological orientation, and obviating the need for presacral dissection. Current studies report that LLS provides anatomical and functional outcomes comparable to laparoscopic sacrocolpopexy, while potentially offering shorter operative times and a more favorable complication profile. Moreover, shorter hospitalization, lower perioperative blood loss, easier postoperative pain management, and superior cosmetic results have been described as additional clinical advantages supporting the adoption of LLS (4) Recent systematic reviews indicate that LLS achieves high anatomical and functional success rates with low rates of mesh erosion and reoperation (5). As an innovative approach, vaginal natural orifice transluminal endoscopic surgery (vNOTES) has recently emerged as a new endoscopic platform for POP repair, enabling the transvaginal application of various apical support strategies such as hysterectomy, uterosacral suspension, sacrospinous fixation, and lateral suspension (6).

The vNOTES approach has become an attractive option particularly for younger, sexually active women who wish to preserve their uterus, as it avoids abdominal incisions and offers advantages such as reduced postoperative pain, decreased analgesic requirements, shorter hospital stay, and superior cosmetic outcomes. Randomized and prospective studies comparing vNOTES with laparoscopy have reported significantly lower postoperative pain, faster mobilization, and earlier discharge in favor of vNOTES (7, 8). In the field of POP surgery, the increasing number of clinical series evaluating vNOTES in recent years suggests that the technique has evolved beyond being merely a hysterectomy platform and is now a feasible option for uterine-sparing prolapse repair. Furthermore, in uterine-preserving POP surgery, vNOTES may offer functional advantages by restoring the vaginal axis in a more physiological manner and allowing mesh placement and peritoneal closure with endoscopic precision.

However, studies combining vNOTES with lateral suspension remain in their early stages. Comparative data between laparoscopic lateral suspension and vNOTES lateral suspension particularly from the perspective of uterine-sparing surgery are scarce. Thus, comparing these two minimally invasive approaches in terms of anatomical success, functional outcomes, complication profiles, recovery dynamics, cosmetic results, and patient satisfaction represents a relevant gap in the current literature. Notably, as the trend toward uterine preservation continues to grow, addressing this gap has implications not only for clinical decision-making, but also for patient preference and quality of-life oriented modern pelvic floor surgery.

## Methods

### Study design and setting

This study was conducted as a prospective observational cohort at a tertiary care center in Diyarbakır, Türkiye. The hospital is a tertiary referral center for urogynecologic surgery and serves as a high-volume hub for advanced pelvic floor procedures. Within our institution, minimally invasive pelvic organ prolapse surgery includes both laparoscopic lateral suspension and vaginal natural orifice transluminal endoscopic surgery (vNOTES) lateral suspension techniques. In both surgical groups, the same lightweight macroporous synthetic polypropylene mesh was used to standardize mesh characteristics between the two surgical approaches. The mesh arms were positioned laterally without fascial fixation and subsequently retroperitonealized by peritoneal closure to minimize mesh exposure and ensure anatomical support. During the preoperative assessment period, surgical candidates were counseled, and the choice of surgical approach was determined based on anatomical considerations, surgeon expertise, and patient preference. No randomization was performed. Following surgery, all patients were monitored according to standardized institutional protocols. Perioperative variables were recorded in real time, and postoperative clinical data were prospectively entered into an electronic data registry. Follow-up evaluations were scheduled at 3 and 6 months, during which Pelvic Organ Prolapse Quantification (POP-Q) measurements and functional questionnaires (POPDI-6, UDI-6, and CRADI-8) were administered.

### Study population and eligibility criteria

**Figure 1:**
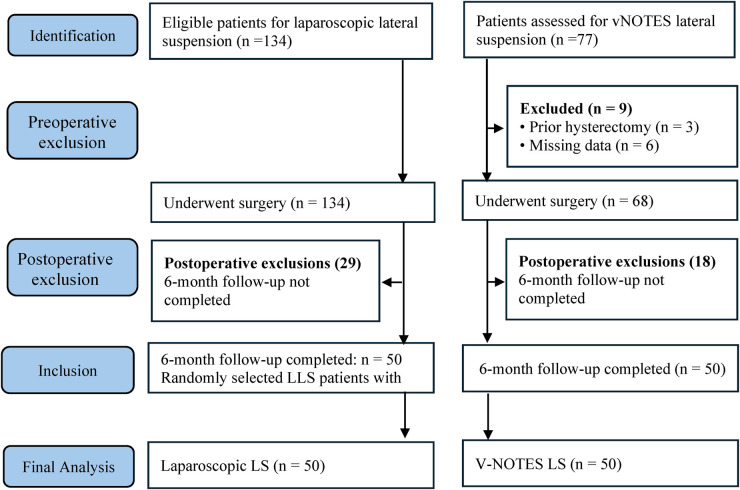
Flow diagram of patient screening, eligibility, allocation, and follow-up for the vNOTES and laparoscopic lateral suspension arms.

Women who underwent uterus-preserving prolapse surgery using either vNOTES lateral suspension or laparoscopic lateral suspension (LLS) during the study period were evaluated. The patient selection and follow-up process are illustrated in Figure 1. In total, 77 patients were assessed for vNOTES and 134 for laparoscopic lateral suspension. After exclusion of nine patients due to prior hysterectomy (*n* = 3) or missing data (*n* = 6), 68 patients underwent vNOTES lateral suspension. During postoperative follow-up, 18 vNOTES and 29 laparoscopic patients did not complete the prespecified 6-month follow-up and were excluded. The first 50 vNOTES patients who completed the 6-month follow-up were included in the study. For comparison, 50 laparoscopic lateral suspension patients with completed follow-up were randomly selected from the institutional database. Eligible participants had symptomatic anterior–apical pelvic organ prolapse (POP-Q stage ≥ II) and were older than 18 years. The final analysis therefore included 100 patients (vNOTES *n* = 50; LLS *n* = 50). Postoperative POP-Q measurements and functional questionnaires were assessed, whenever possible, by clinicians who were not involved in the surgical procedures. Due to the clinical nature of follow-up examinations, complete blinding of the evaluators to the surgical technique was not always feasible; however, all assessments were performed according to standardized institutional protocols to minimize potential observer bias.

### Surgical techniques

#### vNOTES lateral suspension technique

In the vNOTES group, transvaginal endoscopic access was obtained via anterior colpotomy. After establishing the vNOTES platform, the peritoneal cavity was accessed endoscopically. Dissection was performed to create appropriate lateral pathways, and a lightweight macroporous polypropylene mesh was positioned to provide apical support. The mesh arms were directed laterally without fascial fixation, and the peritoneum was closed endoscopically to retroperitonealize the mesh.

#### Laparoscopic lateral suspension technique

In the laparoscopic group, a supraumbilical camera port and two accessory trocars were placed. Peritoneal dissection was extended laterally, and apical support was achieved by directing bilateral mesh arms through retroperitoneal tunnels toward the abdominal wall. No fascial fixation was applied. The mesh was subsequently retroperitonealized by peritoneal closure. All procedures were performed by surgeons experienced in both laparoscopic lateral suspension and vNOTES prolapse surgery. All procedures were performed by surgeons experienced in minimally invasive pelvic floor surgery who had completed the learning curve for vNOTES lateral suspension prior to the initiation of the study.Preoperative evaluation included POP-Q assessment, and a uterus-preserving approach was adopted in all cases. In the laparoscopic group, a supraumbilical camera port and two accessory trocars were placed. Peritoneal dissection was extended laterally, and apical support was achieved by directing the bilateral mesh arms through retroperitoneal tunnels toward the abdominal wall. No fascial fixation or tensioned suturing was applied, in order to reduce the risk of cutaneous dimpling, skin retraction, and postoperative pain. The mesh was retroperitonealized by peritoneal closure. In the vNOTES group, transvaginal endoscopic access was obtained via anterior colpotomy, and mesh arms were similarly directed laterally without the need for abdominal trocar placement or fascial incisions. By avoiding abdominal access, the procedure eliminated trocar-, Veress-, and camera-related entry complications and obviated the need to advance the mesh through the abdominal wall from outside the body. Uterine apical support was achieved without fascial fixation, and the peritoneum was closed endoscopically. Mesh characteristics, dissection planes, and peritoneal closure techniques were standardized between groups, and concomitant compartment repairs were performed when clinically indicated.

#### Ethical considerations

The study protocol was reviewed and approved by the Clinical Research Ethics Committee of the University of Health Sciences Gazi Yaşargil Training and Research Hospital, Diyarbakır, Türkiye (Approval Date: 19.09.2024; Approval No.: 640). As a prospective cohort study, written informed consent was obtained from all participants prior to surgical intervention and data collection. Patient confidentiality and data protection were ensured in accordance with the Declaration of Helsinki and applicable national regulations.

#### Statistical analysis

Continuous variables were summarized as mean ± standard deviation (SD), whereas categorical variables were presented as frequencies and percentages. Between-group comparisons of continuous variables were performed using Student's t-test or the Mann–Whitney U test, depending on distributional characteristics. Within-group comparisons between 3- and 6-month follow-up measurements were performed using paired t-tests. Categorical variables were compared using Pearson's x^2^ test or Fisher's exact test when appropriate. A two-tailed *p*-value <0.05 was considered statistically significant. All statistical analyses were conducted using IBM SPSS Statistics version 26 (IBM Corp., Armonk, NY, USA). Radar plot visualization was used to illustrate functional outcome domains across follow-up time points.

## Results

A total of 100 women completed the 6-month follow-up and were included in the final analysis, with 50 undergoing vNOTES lateral suspension and 50 treated by laparoscopic lateral suspension (LLS). The flow of patient selection, eligibility, and follow-up is shown in Figure 1 The two groups demonstrated similar baseline demographic and clinical profiles. The mean age was 46.0 ± 10.8 years in the vNOTES group and 50.6 ± 14.6 years in the LLS group (*p* = 0.12). Body mass index was also comparable between groups (27.7 ± 2.9 vs. 27.1 ± 2.6 kg/m^2^; *p* = 0.35). Gravidity did not differ significantly (*p* = 0.66), and approximately half of the patients in each cohort reported at least one medical comorbidity (48% vs. 50%; *p* = 0.84). A history of prior prolapse surgery was more frequent in the vNOTES cohort (76% vs. 62%), although this difference did not reach statistical significance (*p* = 0.14). Previous abdominal surgery was reported in 30% of vNOTES cases and 24% of LLS cases (*p* = 0.64). Overall, no significant differences were observed across baseline demographic or clinical characteristics (all *p* > 0.05). Detailed baseline data are provided in Table 1.

**Table 1:** Baseline demographic and clinical characteristics of patients undergoing V-NOTES lateral suspension versus laparoscopic lateral suspension.

Indicators	V-NOTES LS (*n* = 50)	Laparoscopic LS (*n* = 50)	*p*-value
Age (years), mean ± SD	46.0 ± 10.8	50.6 ± 14.6	0.12
BMI (kg/m2), mean ± SD	27.74 ± 2.88	27.14 ± 2.56	0.35
Gravida, mean ± SD	4.30 ± 1.11	4.22 ± 0.95	0.66
Premenopausal, *n* (%)	27 (54%)	22 (44%)	0.42
Comorbidity, *n* (%)	24 (48%)	25 (50%)	0.84
Prior prolapse surgery, *n* (%)	38 (76%)	31 (62%)	0.14
Previous abdominal surgery, *n* (%)	15 (30%)	12 (24%)	0.64

Values are mean ± SD or *n* (%). Mann–Whitney U and Chi-square tests were used.

V-NOTES, vaginal natural orifice transluminal endoscopic surgery; LS, lateral suspension; BMI, body mass index.

All procedures were completed successfully. Perioperative parameters differed notably between the two surgical approaches. The mean operative time was significantly shorter in the vNOTES group compared with the LLS group (60.36 ± 11.17 vs. 90.66 ± 8.40 min; *p* < 0.001). Consistently, patients who underwent vNOTES experienced a shorter postoperative hospital stay (1.32 ± 0.59 vs. 1.98 ± 0.65 days; *p* < 0.001). These findings indicate that the vNOTES approach provides significantly greater perioperative efficiency, as demonstrated by the shorter operative time and reduced length of hospital stay compared with laparoscopic lateral suspension. No significant difference was observed in hemoglobin drop between groups (1.63 ± 1.59 vs. 1.54 ± 0.61 g/dL; *p* = 0.71). Early postoperative complications were infrequent in both cohorts and did not differ statistically (2% vs. 12%; *p* = 0.11). No bladder injury occurred in the vNOTES group, whereas a single bladder perforation was reported in the LLS group. In addition, one case of abdominal hematoma occurred in each group. Detailed perioperative outcomes are provided in Table 2.

**Table 2:** Intraoperative and perioperative outcomes in the V-NOTES and laparoscopic lateral suspension (LS) groups.

Indicators	V-NOTES LS (*n* = 50)	Laparoscopic LS (*n* = 50)	*p*-value
Hemoglobin drop (g/dL)	1.63 ± 1.59	1.54 ± 0.61	0.71
Operative time (min)	60.36 ± 11.17	90.66 ± 8.40	<0.001
Length of hospital stay (days)	1.32 ± 0.59	1.98 ± 0.65	<0.001
Recurrence	0 (0%)	4 (8%)	0.12
Bladder perforation	0 (0%)	1 (2%)	1.00
Abdominal hematoma	1 (2%)	1 (2%)	1.00

Values are mean ± SD or *n* (%). Independent t-test and Fisher's exact test were used (*p* < 0.05).

V-NOTES, vaginal natural orifice transluminal endoscopic surgery; LS, lateral suspension; CI, confidence interval; Hb, hemoglobin; Min, minutes.

Both surgical approaches provided satisfactory anatomical correction of anterior–apical prolapse at the 3- and 6-month follow-up visits. In the vNOTES group, Ba and Bp measurements demonstrated sustained postoperative improvement, with a significant additional gain observed in Bp between the 3- and 6-month assessments (*p* < 0.01). No significant interval change was noted in Ba or C (*p* > 0.05). Total vaginal length remained preserved throughout follow-up in the vNOTES cohort. In the LLS group, POP-Q parameters similarly indicated maintenance of anterior and apical support over time, with no significant within-group differences observed for Ba, C, Bp, or TVL between 3 and 6 months (all *p* > 0.05). Between-group comparisons favored vNOTES for anterior and posterior compartment positioning, as Ba and Bp values were significantly lower in the vNOTES cohort at both time points (*p* < 0.001 for both comparisons). No significant differences were detected for apical point C or total vaginal length between groups. Detailed POP-Q data are provided in Table 3.Both procedures resulted in clinically meaningful improvements in pelvic floor symptom scores during the postoperative period. In the vNOTES group, bowel-related symptoms measured by CRADI-8 showed additional improvement from the 3- to the 6-month assessment (*p* = 0.05). Urinary (UDI-6) and prolapse-related symptom scores remained improved after surgery, without further interval change. In the LLS cohort, no significant within-group differences were observed for POPDI-6, CRADI-8, or UDI-6 between 3 and 6 months (all *p* > 0.05). Between-group comparisons indicated a lower functional symptom burden in the vNOTES cohort. Prolapse-related symptom scores (POPDI-6) were significantly lower in the vNOTES group at both 3 and 6 months (*p* = 0.03 and *p* = 0.006, respectively). Urinary symptom scores (UDI-6) were likewise lower in the vNOTES cohort at both time points (*p* = 0.003 and *p* = 0.005). Bowel-related symptoms showed a significant group difference at the 6-month evaluation, with lower CRADI-8 scores in the vNOTES group (*p* = 0.001). Detailed functional data are presented in Table 3.

**Table 3:** Comparison of POP-Q and functional outcomes between V-NOTES and laparoscopic lateral suspension at 3 and 6 months postoperatively.

Indicators	V-NOTES LS (mean ± SD)		p	Laparoscopic LS (mean ± SD)		p	*p*-value between group	
	3-month	6-month		3-month	6-month		3-month	6-month
Ba (cm)	−2.81 ± 0.3	−2.78 ± 0.3	0.21	−2.49 ± 0.4	−2.42 ± 0.5	0.4	< 0.001	< 0.001
C (cm)	−5.95 ± 0.5	−5.90 ± 0.5	0.56	−5.91 ± 0.4	−5.79 ± 0.3	0.2	0.67	0.17
Bp(cm)	−2.53 ± 0.78	−2.90 ± 0.33	< 0.01	−2.20 ± 0.5	−2.33 ± 0.4	0.2	0.01	< 0.001
TVL (cm)	7.86 ± 0.6	7.82 ± 0.6	0.5	7.53 ± 0.5	7.69 ± 0.5	0.4	0.0036	0.24
POPDI-6	4.68 ± 4.03	4.84 ± 3.12	0.24	6.44 ± 3.77	6.36 ± 3.05	0.5	0.03	0.006
CRADI-8	4.69 ± 2.74	2.96 ± 2.22	0.05	5.46 ± 2.49	4.76 ± 3.02	0.53	0.15	0.001
UDI-6	3.71 ± 3.11	5.09 ± 3.17	0.001	5.66 ± 3.15	6.54 ± 3.26	< 0.01	0.003	0.005

Values are mean ± SD or *n* (%). Between-group comparisons were performed using t-tests for continuous variables and Fisher's exact tests for categorical variables.

Ba, anterior vaginal point; Bp, posterior vaginal point; C, cervix/apical point; TVL, total vaginal length; POPDI-6, pelvic organ prolapse distress inventory-6; CRADI-8, colorectal-anal distress inventory-8; UDI-6, urinary distress inventory-6; PISQ-12, pelvic organ prolapse/urinary incontinence sexual questionnaire-12; V-NOTES, vaginal natural orifice transluminal endoscopic surgery; LS, lateral suspension.

To account for potential confounding factors, an additional multivariable linear regression analysis was performed including age, body mass index (BMI), and gravidity as covariates (Table 4). After adjustment for these variables, surgical technique remained independently associated with operative time. Specifically, the vNOTES approach was associated with a significantly shorter operative time compared with laparoscopic lateral suspension (*β* = −31.9 min, 95% CI −35.7 to −28.1, *p* < 0.001). Age and BMI were also independently related to operative time, suggesting that patient characteristics may have a modest influence on procedural duration. In contrast, gravidity was not significantly associated with operative time in the adjusted model. Overall, these findings indicate that the shorter operative time observed in the vNOTES group persisted even after controlling for relevant baseline characteristics.

**Table 4:** Multivariable linear regression analysis evaluating factors associated with operative time.

Indicators	*β* (95% CI)	*p*-value
vNOTES vs LLS	−31.9 (−35.7 to −28.1)	<0.001
Age	−0.19 (−0.34 to −0.04)	0.014
BMI	0.98 (0.24 to 1.72)	0.010
Gravidity	1.46 (−0.46 to 3.38)	0.134

Values are presented as β coefficients with 95% confidence intervals (CI). Multivariable linear regression adjusted for age, BMI, and gravidity was performed. Surgical technique was coded as vNOTES vs LLS.

**Figure 2:**
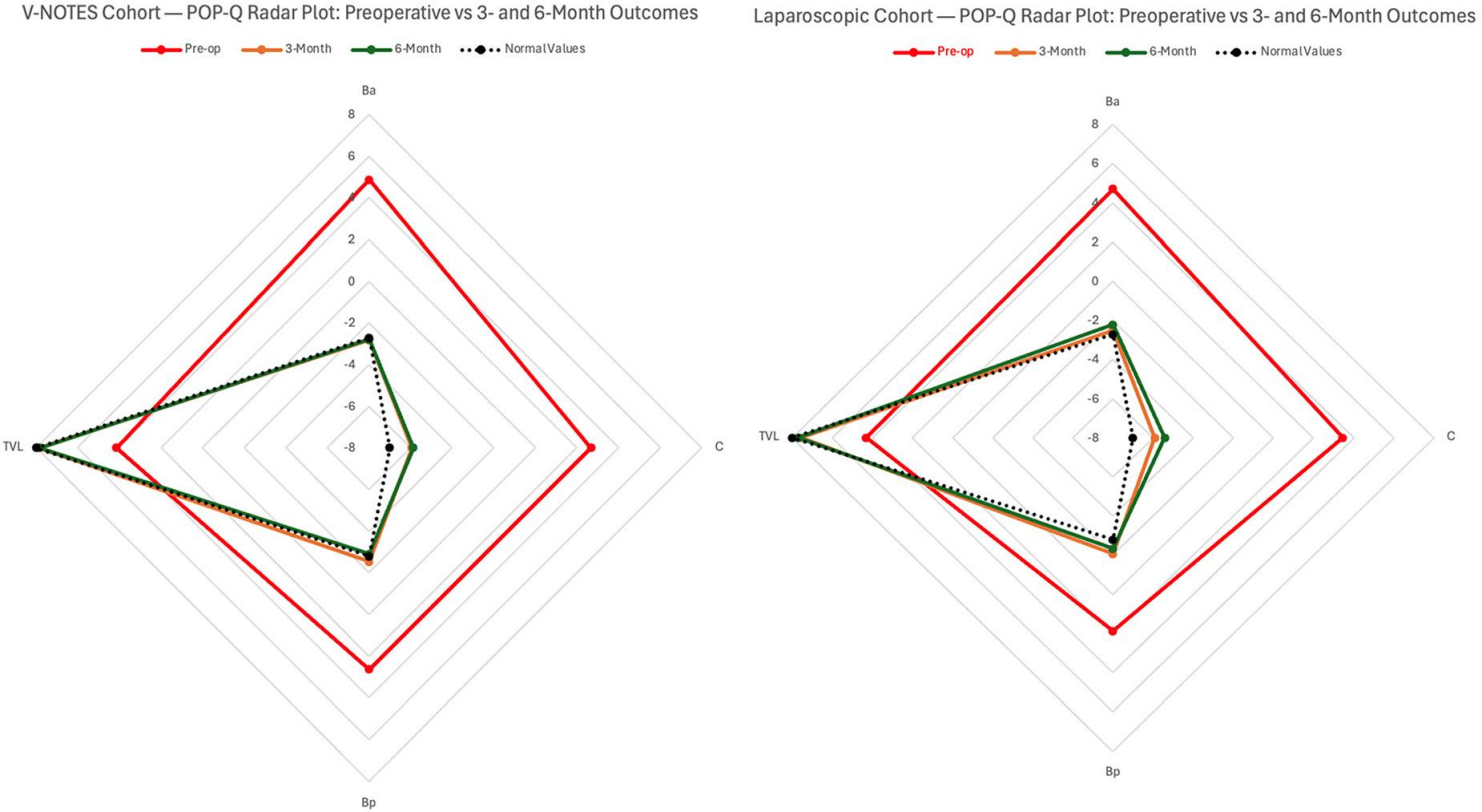
Radar plot comparison of POP-Q parameters at 3- and 6-month follow-Up after V-NOTES versus laparoscopic lateral suspension.

The radar plot provides a visual comparison of the postoperative positioning of the POP-Q parameters Ba, Bp, C, and total vaginal length (TVL) relative to a reference contour shown in black, which represents the ideal or expected anatomical configuration. Following surgery, both groups demonstrated a noticeable shift of these parameters toward the reference contour, suggesting that each approach contributed to anatomical restoration of the anterior–apical compartment. Although improvement was observed in both cohorts, the vNOTES group exhibited a more uniform and symmetrical approximation toward the reference contour across all four POP-Q points, while the trajectory in the laparoscopic cohort appeared less consistent and showed greater variability between parameters. Taken together, the visual information conveyed by the radar plot reinforces the numerical POP-Q findings reported in Table 2 and supports the interpretation that both procedures were effective in improving pelvic support. The corresponding radar visualization is presented in Figure 2.

## Limitations

This study has several limitations. It was a non-randomized, single-center cohort with a modest sample size and 6-month follow-up, which may limit generalizability and underpower small functional differences. Long-term durability beyond 6 months remains unknown. Larger multicenter randomized trials with extended follow-up are needed to validate these findings. Another limitation of this study is that concomitant pelvic floor repair procedures, such as posterior compartment repair (rectocele repair), were not systematically recorded in the dataset. Therefore, the potential influence of additional pelvic floor interventions on postoperative outcomes could not be evaluated. In addition, the surgical approach was determined based on patient preference and surgeon expertise rather than randomization, which may introduce potential selection bias. Furthermore, the relatively small sample size may limit the ability to detect subtle differences in functional outcomes or rare complications. Because of the 6-month follow-up duration, late mesh-related complications such as erosion or contraction could not be adequately assessed. In addition, the relatively short follow-up period may limit the ability to fully assess late mesh-related complications such as mesh erosion, contraction, or exposure.

## Strengths

Strengths of this study include its prospective design, standardized surgical techniques, use of validated functional questionnaires, and combined assessment of anatomical, urinary, and sexual function outcomes. The inclusion of a comparison group with similar follow-up windows and the use of radar visualization for POP-Q parameters further enhance the methodological robustness and clinical interpretability of the results.

## Discussion

The present study compared vNOTES lateral suspension with laparoscopic lateral suspension for anterior–apical pelvic organ prolapse and found that both uterus-preserving techniques provided favorable anatomical correction at the 6-month follow-up. From a clinical perspective, the two approaches achieved comparable improvements in POP-Q measurements within the apical and anterior compartments, while the vNOTES cohort demonstrated a more uniform approximation toward the ideal anatomical configuration on radar assessment. Importantly, both groups experienced functional symptom improvement, which aligns with the clinical expectation that restoration of apical support can optimize the vaginal axis and length and thereby alleviate pelvic floor symptoms. However, the perioperative profile clearly differentiated the two techniques. Although vNOTES offers several perioperative advantages, the technique requires familiarity with transvaginal endoscopic orientation and may involve an initial learning curve for surgeons adopting this approach. Previous studies have suggested that surgical proficiency in vNOTES procedures is generally achieved after approximately 20 cases (9). In our study, all procedures were performed in a tertiary referral center with extensive experience in minimally invasive pelvic floor surgery. The surgeons performing the operations were the authors of the study and had already completed more than 50 vNOTES lateral suspension procedures before the initiation of the study. Therefore, the study was conducted after the learning phase had largely been overcome, minimizing the potential impact of the learning curve on the reported outcomes. Both operative time and length of hospital stay were significantly shorter in the vNOTES cohort, which is clinically relevant in terms of patient recovery and healthcare resource use. These findings are consistent with recent reports indicating that vNOTES can improve surgical efficiency by enhancing visualization in the deep pelvis and eliminating abdominal trocar morbidity. Issat et al. similarly showed reduced operative time and shorter hospitalization with vNOTES compared with conventional minimally invasive approaches in gynecologic surgery. Comparable results have been reported in apical prolapse cohorts, where vNOTES lateral suspension demonstrated shorter admissions and fewer perioperative complications than laparoscopic lateral suspension (10). Comparable conclusions have also been drawn in studies evaluating vNOTES for apical suspension procedures, where faster recovery and improved cosmetic satisfaction have been reported (11).

Our findings are broadly consistent with the comparative study by Bulutlar et al., which also reported shorter operative time and faster postoperative recovery with vNOTES lateral suspension compared with laparoscopic lateral suspension. In the present study, this perioperative advantage was accompanied by favorable anterior and posterior compartment outcomes, with lower Ba and Bp values in the vNOTES group, while apical support at point C remained comparable between groups. One possible explanation is that the transvaginal access provided by vNOTES allows more direct visualization of pelvic floor structures and facilitates mesh placement along a more physiological vaginal plane. This may enable a more balanced distribution of mesh tension across the anterior and posterior compartments. In contrast, during laparoscopic lateral suspension the mesh is positioned from an abdominal perspective, which may influence tension distribution across pelvic compartments differently. Nevertheless, the similar results observed at point C suggest that both techniques remain effective in restoring apical support. This pattern suggests that the benefit of vNOTES may be more pronounced in compartmental support and early recovery rather than in apical descent alone.

Over the last decade, both laparoscopic lateral suspension (LLS) and vNOTES lateral suspension have emerged as validated minimally invasive approaches for anterior–apical prolapse repair that allow uterine preservation, physiologic restoration of the vaginal axis, and low mesh-related complication rates. Contemporary prospective data and systematic reviews report high anatomical success, low reoperation rates, and favorable perioperative recovery patterns with both techniques, supporting their integration into modern uterus-sparing pelvic floor surgery (12–14). This pattern has been described in contemporary reconstructive vNOTES series, where improvements in lower urinary tract symptoms and patient-reported functional scales have also been documented (15, 16). An additional point that deserves consideration is the increase in UDI-6 scores between the 3- and 6-month evaluations in the vNOTES group. Although the overall between-group comparison continued to favor vNOTES, this within-group increase may reflect the emergence of *de novo* urinary symptoms after prolapse reduction, including possible stress urinary incontinence that becomes more apparent once the prolapse is anatomically corrected. Another possible explanation is that restoration of anterior compartment support may alter lower urinary tract dynamics in a subset of patients. Although the study was not specifically designed to distinguish these mechanisms, this finding should be interpreted cautiously and merits further investigation in studies with longer follow-up and more detailed urogynecologic assessment. Perioperatively, vNOTES was associated with shorter operative time and shorter postoperative hospitalization compared with LLS in our study, results that parallel previously reported shorter length of stay and faster recovery profiles in minimally invasive vNOTES cohorts (17). Another point requiring cautious interpretation is the higher proportion of patients with prior prolapse surgery in the vNOTES group compared with the laparoscopic group (76% vs. 62%). Although this difference was not statistically significant, it may still be clinically relevant, since prior prolapse surgery can alter tissue planes, surgical dissection, and operative complexity. Therefore, this imbalance should be considered a potential confounding factor when interpreting the comparative outcomes. Importantly, no mesh exposure or erosion was observed in our cohort, echoing the reassuring early mesh safety profiles reported in contemporary vNOTES apical suspension cohorts. Historically, transvaginal mesh implantation for pelvic organ prolapse repair has been associated with mesh exposure rates ranging from approximately 5% to 15%, which has raised significant safety concerns and led to regulatory restrictions in several countries. In contrast, the lateral suspension technique used in our study places the mesh away from the vaginal epithelium and avoids direct transvaginal mesh implantation. This anatomical configuration may contribute to the lower mesh-related complication rates reported in contemporary minimally invasive apical suspension procedures (18). Another potential explanation for the absence of mesh exposure in the present study may relate to the surgical configuration of the mesh. In vNOTES lateral suspension, the mesh is placed entirely within the intraperitoneal compartment and covered by peritoneal closure performed under endoscopic visualization. This approach theoretically minimizes direct contact between the prosthetic material and the vaginal epithelium, which may reduce the risk of mesh exposure or erosion compared with transvaginal mesh placement. Previous reports evaluating minimally invasive apical suspension techniques have also suggested that intraperitoneal mesh positioning and peritonealization may contribute to improved mesh safety profiles (19).

Taken together, these findings suggest that vNOTES may offer a uniquely favorable balance between efficient recovery, durable symptom relief, and uterus preservation, without compromising anatomical support or safety. While longer-term data remain necessary, the early postoperative trajectory observed in our cohort aligns with the evolving clinical role of vNOTES within minimally invasive apical prolapse repair.

## Conclusion

In this prospective cohort, both vNOTES lateral suspension and laparoscopic lateral suspension provided effective uterus-preserving repair for anterior–apical pelvic organ prolapse, achieving favorable anatomical support and meaningful symptom relief within six months. Perioperative recovery was notably more efficient in the vNOTES cohort, with shorter operative time and hospitalization, while functional improvements in urinary and prolapse-related domains were more pronounced and sustained. Importantly, no mesh exposure or erosion was observed in either group during follow-up, supporting the early safety of mesh-based apical suspension in appropriately selected patients. These findings suggest that vNOTES lateral suspension may represent an attractive minimally invasive alternative for women seeking uterus-sparing prolapse repair, offering a balanced profile of efficient recovery, functional benefit, and anatomical success without compromising safety. As the clinical demand for uterine preservation continues to rise, comparative data such as ours may help inform individualized surgical decision-making. Longer-term and larger-scale studies are warranted to evaluate durability, mesh performance over time, and patient-reported outcomes beyond the early postoperative period.

## Data Availability

The raw data supporting the conclusions of this article will be made available by the authors, without undue reservation.
